# A review of the quantitative effectiveness evidence synthesis methods used in public health intervention guidelines

**DOI:** 10.1186/s12889-021-10162-8

**Published:** 2021-02-03

**Authors:** Ellesha A. Smith, Nicola J. Cooper, Alex J. Sutton, Keith R. Abrams, Stephanie J. Hubbard

**Affiliations:** Department of Health Sciences, University of Leicester, Lancaster Road, Leicester, UK

**Keywords:** Meta-analysis, Network meta-analysis, Systematic review, Public health, Decision making, Complex interventions, Evidence synthesis

## Abstract

**Background:**

The complexity of public health interventions create challenges in evaluating their effectiveness. There have been huge advancements in quantitative evidence synthesis methods development (including meta-analysis) for dealing with heterogeneity of intervention effects, inappropriate ‘lumping’ of interventions, adjusting for different populations and outcomes and the inclusion of various study types. Growing awareness of the importance of using all available evidence has led to the publication of guidance documents for implementing methods to improve decision making by answering policy relevant questions.

**Methods:**

The first part of this paper reviews the methods used to synthesise quantitative effectiveness evidence in public health guidelines by the National Institute for Health and Care Excellence (NICE) that had been published or updated since the previous review in 2012 until the 19th August 2019.The second part of this paper provides an update of the statistical methods and explains how they address issues related to evaluating effectiveness evidence of public health interventions.

**Results:**

The proportion of NICE public health guidelines that used a meta-analysis as part of the synthesis of effectiveness evidence has increased since the previous review in 2012 from 23% (9 out of 39) to 31% (14 out of 45). The proportion of NICE guidelines that synthesised the evidence using only a narrative review decreased from 74% (29 out of 39) to 60% (27 out of 45).An application in the prevention of accidents in children at home illustrated how the choice of synthesis methods can enable more informed decision making by defining and estimating the effectiveness of more distinct interventions, including combinations of intervention components, and identifying subgroups in which interventions are most effective.

**Conclusions:**

Despite methodology development and the publication of guidance documents to address issues in public health intervention evaluation since the original review, NICE public health guidelines are not making full use of meta-analysis and other tools that would provide decision makers with fuller information with which to develop policy. There is an evident need to facilitate the translation of the synthesis methods into a public health context and encourage the use of methods to improve decision making.

**Supplementary Information:**

The online version contains supplementary material available at (10.1186/s12889-021-10162-8).

## Background

To make well-informed decisions and provide the best guidance in health care policy, it is essential to have a clear framework for synthesising good quality evidence on the effectiveness and cost-effectiveness of health interventions. There is a broad range of methods available for evidence synthesis. Narrative reviews provide a qualitative summary of the effectiveness of the interventions. Meta-analysis is a statistical method that pools evidence from multiple independent sources [1]. Meta-analysis and more complex variations of meta-analysis have been extensively applied in the appraisals of clinical interventions and treatments, such as drugs, as the interventions and populations are clearly defined and tested in randomised, controlled conditions. In comparison, public health studies are often more complex in design, making synthesis more challenging [2].

Many challenges are faced in the synthesis of public health interventions. There is often increased methodological heterogeneity due to the inclusion of different study designs. Interventions are often poorly described in the literature which may result in variation within the intervention groups. There can be a wide range of outcomes, whose definitions are not consistent across studies. Intermediate, or surrogate, outcomes are often used in studies evaluating public health interventions [3]. In addition to these challenges, public health interventions are often also complex meaning that they are made up of multiple, interacting components [4]. Recent guidance documents have focused on the synthesis of complex interventions [2, 5, 6]. The National Institute for Health and Care Excellence (NICE) guidance manual provides recommendations across all topics that are covered by NICE and there is currently no guidance that focuses specifically on the public health context.

### Research questions

A methodological review of NICE public health intervention guidelines by Achana et al. (2014) found that meta-analysis methods were not being used [3]. The first part of this paper aims to update and compare, to the original review, the meta-analysis methods being used in evidence synthesis of public health intervention appraisals.

The second part of this paper aims to illustrate what methods are available to address the challenges of public health intervention evidence synthesis. Synthesis methods that go beyond a pairwise meta-analysis are illustrated through the application to a case study in public health and are discussed to understand how evidence synthesis methods can enable more informed decision making.

The third part of this paper presents software, guidance documents and web tools for methods that aim to make appropriate evidence synthesis of public health interventions more accessible. Recommendations for future research and guidance production that can improve the uptake of these methods in a public health context are discussed.

## Update of NICE public health intervention guidelines review

### NICE guidelines

The National Institute for Health and Care Excellence (NICE) was established in 1999 as a health authority to provide guidance on new medical technologies to the NHS in England and Wales [7]. Using an evidence-based approach, it provides recommendations based on effectiveness and cost-effectiveness to ensure an open and transparent process of allocating NHS resources [8]. The remit for NICE guideline production was extended to public health in April 2005 and the first recommendations were published in March 2006. NICE published ‘Developing NICE guidelines: the manual’ in 2006, which has been updated since, with the most recent in 2018 [9]. It was intended to be a guidance document to aid in the production of NICE guidelines across all NICE topics. In terms of synthesising quantitative evidence, the NICE recommendations state: ‘meta-analysis may be appropriate if treatment estimates of the same outcome from more than 1 study are available’ and ‘when multiple competing options are being appraised, a network meta-analysis should be considered’. The implementation of network meta-analysis (NMA), which is described later, as a recommendation from NICE was introduced into the guidance document in 2014, with a further update in 2018.

### Background to the previous review

The paper by Achana et al. (2014) explored the use of evidence synthesis methodology in NICE public health intervention guidelines published between 2006 and 2012 [3]. The authors conducted a systematic review of the methods used to synthesise quantitative effectiveness evidence within NICE public health guidelines. They found that only 23% of NICE public health guidelines used pairwise meta-analysis as part of the effectiveness review and the remainder used a narrative summary or no synthesis of evidence at all. The authors argued that despite significant advances in the methodology of evidence synthesis, the uptake of methods in public health intervention evaluation is lower than other fields, including clinical treatment evaluation. The paper concluded that more sophisticated methods in evidence synthesis should be considered to aid in decision making in the public health context [3].

### Methods

The search strategy used in this paper was equivalent to that in the previous paper by Achana et al. (2014)[3]. The search was conducted through the NICE website (https://www.nice.org.uk/guidance) by searching the ‘Guidance and Advice List’ and filtering by ‘Public Health Guidelines’ [10]. The search criteria included all guidance documents that had been published from inception (March 2006) until the 19th August 2019. Since the original review, many of the guidelines had been updated with new documents or merged. Guidelines that remained unchanged since the previous review in 2012 were excluded and used for comparison.

The guidelines contained multiple documents that were assessed for relevance. A systematic review is a separate synthesis within a guideline that systematically collates all evidence on a specific research question of interest in the literature. Systematic reviews of quantitative effectiveness, cost-effectiveness evidence and decision modelling reports were all included as relevant. Qualitative reviews, field reports, expert opinions, surveillance reports, review decisions and other supporting documents were excluded at the search stage.

Within the reports, data was extracted on the types of review (narrative summary, pairwise meta-analysis, network meta-analysis (NMA), cost-effectiveness review or decision model), design of included primary studies (randomised controlled trials or non-randomised studies, intermediate or final outcomes, description of outcomes, outcome measure statistic), details of the synthesis methods used in the effectiveness evaluation (type of synthesis, fixed or random effects model, study quality assessment, publication bias assessment, presentation of results, software). Further details of the interventions were also recorded, including whether multiple interventions were lumped together for a pairwise comparison, whether interventions were complex (made up of multiple components) and details of the components. The reports were also assessed for potential use of complex intervention evidence synthesis methodology, meaning that the interventions that were evaluated in the review were made up of components that could potentially be synthesised using an NMA or a component NMA [11]. Where meta-analysis was not used to synthesis effectiveness evidence, the reasons for this was also recorded.

### Results

#### Search results and types of reviews

There were 67 NICE public health guidelines available on the NICE website. A summary flow diagram describing the literature identification process and the list of guidelines and their reference codes are provided in Additional files 1 and 2. Since the previous review, 22 guidelines had not been updated. The results from the previous review were used for comparison to the 45 guidelines that were either newly published or updated.

The guidelines consisted of 508 documents that were assessed for relevance. Table 1 shows which types of relevant documents were available in each of the 45 guidelines. The median number of relevant articles per guideline was 3 (minimum = 0, maximum = 10). Two (4%) of the NICE public health guidelines did not report any type of systematic review, cost-effectiveness review or decision model (NG68, NG64) that met the inclusion criteria. 167 documents from 43 NICE public health guidelines were systematic reviews of quantitative effectiveness, cost-effectiveness or decision model reports and met the inclusion criteria.

**Table 1:** Contents of the NICE public health intervention guidelines

Reference code	Systematic review of effectiveness (Narrative review)	Systematic review of effectiveness (At least one meta-analysis)	Systematic review of effectiveness (At least one network meta-analysis)	Cost effectiveness review	Decision model
NG105	$\checkmark $	$\checkmark $		$\checkmark $	$\checkmark $
NG102	$\checkmark $	$\checkmark $		$\checkmark $	$\checkmark $
NG103	$\checkmark $	$\checkmark $		$\checkmark $	$\checkmark $
NG90	$\checkmark $			$\checkmark $	$\checkmark $
NG92	$\checkmark $	$\checkmark $		$\checkmark $	$\checkmark $
NG70					$\checkmark $
NG68					
NG64					
NG63	$\checkmark $				
NG60	$\checkmark $			$\checkmark $	
NG58	$\checkmark $	$\checkmark $		$\checkmark $	$\checkmark $
NG55	$\checkmark $	$\checkmark $			
NG48	$\checkmark $	$\checkmark $			$\checkmark $
NG44	$\checkmark $	$\checkmark $		$\checkmark $	$\checkmark $
NG34	$\checkmark $			$\checkmark $	$\checkmark $
NG30	$\checkmark $			$\checkmark $	$\checkmark $
NG32	$\checkmark $			$\checkmark $	$\checkmark $
NG16	$\checkmark $			$\checkmark $	$\checkmark $
NG13	$\checkmark $			$\checkmark $	$\checkmark $
NG6	$\checkmark $			$\checkmark $	
NG7	$\checkmark $				
PH56	$\checkmark $				$\checkmark $
PH55	$\checkmark $			$\checkmark $	$\checkmark $
PH54	$\checkmark $	$\checkmark $		$\checkmark $	
PH53	$\checkmark $	$\checkmark $			$\checkmark $
PH51	$\checkmark $			$\checkmark $	$\checkmark $
PH52	$\checkmark $			$\checkmark $	
PH50				$\checkmark $	$\checkmark $
PH49	$\checkmark $			$\checkmark $	
PH48	$\checkmark $	$\checkmark $		$\checkmark $	$\checkmark $
PH47	$\checkmark $	$\checkmark $		$\checkmark $	$\checkmark $
PH46	$\checkmark $				
PH45	$\checkmark $			$\checkmark $	$\checkmark $
PH44	$\checkmark $	$\checkmark $			$\checkmark $
PH43	$\checkmark $			$\checkmark $	$\checkmark $
PH41	$\checkmark $			$\checkmark $	$\checkmark $
PH42	$\checkmark $			$\checkmark $	$\checkmark $
PH40	$\checkmark $			$\checkmark $	$\checkmark $
PH39	$\checkmark $				$\checkmark $
PH38	$\checkmark $	$\checkmark $	$\checkmark $	$\checkmark $	$\checkmark $
PH32	$\checkmark $			$\checkmark $	$\checkmark $
PH28	$\checkmark $			$\checkmark $	$\checkmark $
PH21	$\checkmark $			$\checkmark $	$\checkmark $
PH14	$\checkmark $			$\checkmark $	$\checkmark $
PH11	$\checkmark $			$\checkmark $	$\checkmark $
	41 (91%)	14 (31%)	1 (2%)	33 (73%)	34 (76%)

Narrative reviews of effectiveness were implemented in 41 (91%) of the NICE PH guidelines. 14 (31%) contained a review that used meta-analysis to synthesise the evidence. Only one (1%) NICE guideline contained a review that implemented NMA to synthesise the effectiveness of multiple interventions; this was the same guideline that used NMA in the original review and had been updated. 33 (73%) guidelines contained cost-effectiveness reviews and 34 (76%) developed a decision model.

#### Comparison of review types to original review

Table 2 compares the results of the update to the original review and shows that the types of reviews and evidence synthesis methodologies remain largely unchanged since 2012. The proportion of guidelines that only contain narrative reviews to synthesise effectiveness or cost-effectiveness evidence has reduced from 74% to 60% and the proportion that included a meta-analysis has increased from 23% to 31%. The proportion of guidelines with reviews that only included evidence from randomised controlled trials and assessed the quality of individual studies remained similar to the original review.

**Table 2:** Comparison of methods of original review. RCT: randomised controlled trial

Number of guidelines (%)	Original review (39 guidelines)	Updated review (45 guidelines)
No review	1 (3%)	2 (4%)
Narrative review only	29 (74%)	27 (60%)
Meta-analysis	9 (23%)	14 (31%)
Cost effectiveness review	38 (97%)	33 (73%)
Decision model	35 (90%)	34 (76%)
Evidence from RCTs only	2 (5%)	4 (8%)
Study quality assessed	38 (97%)	42 (93%)

#### Characteristics of guidelines using meta-analytic methods

Table 3 details the characteristics of the meta-analytic methods implemented in 24 reviews of the 14 guidelines that included one. All of the reviews reported an assessment of study quality, 12 (50%) reviews included only data from randomised controlled trials, 4 (17%) reviews used intermediate outcomes (e.g. uptake of chlamydia screening rather than prevention of chlamydia (PH3)), compared to the 20 (83%) reviews that used final outcomes (e.g. smoking cessation rather than uptake of a smoking cessation programme (NG92)). 2 (8%) reviews only used a fixed effect meta-analysis, 19 (79%) reviews used a random effects meta-analysis and 3 (13%) did not report which they had used.

**Table 3:** Meta-analytic methods used in the NICE public health intervention appraisals to synthesise the effectiveness evidence

Reference number	Articles	Evidence type	Quality grading of evidence	Includes RCTs only	Final outcome	Description of main outcome	Outcome measure statistic	Type of synthesis	M-A: Fixed or Random (F/R)	Lumping multiple interventions in comparison	Potential intervention components	Details of intervention components	Presentation of results	Assessed publication bias	Software
NG105	Evidence review 1: Multi-agency partnerships	E,CE	$\checkmark $		$\checkmark $	Suicide rate	RR	MA	nr	$\checkmark $			Txt		nr
NG105	Evidence review 4: Information, advice, education and training	E,CE	$\checkmark $		$\checkmark $	Suicide attempts	RR / change in %	MA	nr	$\checkmark $	$\checkmark $	Components that provide information, advice, education for staff or public.	Txt		nr
NG105	Evidence review 6: Reducing access to means	E,CE	$\checkmark $		$\checkmark $	Suicide rate	IRR / difference in number	MA	nr	$\checkmark $	$\checkmark $	Physical barriers, surveillance, encouraging help-seeking.	T		nr
NG102	Evidence review 3: Offering behavioural support to promote health and wellbeing	E, CE, DM	$\checkmark $		$\checkmark $	Weight change	MD	MA	R	$\checkmark $			FP/Txt		RevMan
NG103	Evidence review 2: Increasing flu vaccination uptake in children	E,CE	$\checkmark $			Uptake of vaccinations	RR	MA	R				FP/Txt/T		RevMan
NG103	Evidence review 3: Increasing flu vaccination uptake in clinical risk groups	E,CE	$\checkmark $			Uptake of vaccinations	RR	MA	R				FP/Txt/T		RevMan
NG103	Evidence review 4: Increasing flu vaccination uptake in health and social care staff	E,CE	$\checkmark $			Uptake of vaccinations	RR	MA	R				FP/Txt/T		RevMan
NG92	Non NHS treatments for smoking cessation	E	$\checkmark $	$\checkmark $	$\checkmark $	Smoking cessation	OR	MA	R				FP/Txt		Revman
NG58	Evidence review 3 - The effectiveness and efficiency of service delivery models for health, social care and voluntary and community sector organisations at meeting the needs of people with a severe mental illness who also misuse substances	E	$\checkmark $	$\checkmark $	$\checkmark $	Mental and physical health outcomes (including mortality, recovery and relapse, physical morbidity)	RR	MA	R				FP/Txt		RevMan
NG55	Evidence review 2 - Assessment	E	$\checkmark $		$\checkmark $	Recidivism	Area under curve	MA	R				FP/Txt		R (meta-for)
NG48	Evidence review 1	E	$\checkmark $		$\checkmark $	Plaque index / gingival index	SMD	MA	R	$\checkmark $	$\checkmark $	Assessments of oral health, maintaining access to dental ser- vices, staff training, oral health education, providing oral health resources.	FP/Txt		Stata
NG44	Evidence review 2 - Community engagement 2015 - Brunton	E	$\checkmark $		$\checkmark $	Self-efficacy post test outcome	SMD	MA	R		$\checkmark $	Community engage- ment components: coalitions, collabo- rations, stakeholder involvement, advisory groups or partnerships.	FP/Txt		Stata (metareg)
NG44	Evidence review 3 - Community engagement 2015 - Stokes	E	$\checkmark $	$\checkmark $	$\checkmark $	Behavioural outcomes	SMD	MA	R				FP/Txt	$\checkmark $	Stata (metareg)
Reference number	articles	Evidence type	Quality grading of evidence	Includes RCTs only	Final outcome	Description of main outcome	Outcome measure statistic	Type of synthesis	M-A: Fixed or Random (F/R)	Lumping multiple interventions in comparison	Potential intervention components	Details of intervention components	Presentation of results	Assessed publication bias	Software
PH54	Evidence review 1 - A systematic review and economic evaluation - a short report	E, CE	$\checkmark $	$\checkmark $		Physical activity (self-report or objectively monitored), physical fitness (e.g. maximal oxygen uptake (VO2max), health outcomes (e.g. blood pressure), adverse events (e.g. musculoskeletal injury), and uptake and adherence to ERS.	MD	MA	R		$\checkmark $	Combination of coun- selling, written materi- als, supervised exercise training.	FP/Txt		RevMan
PH53	Evidence review 1a	E	$\checkmark $	$\checkmark $	$\checkmark $	BOCF weight change	MD	MA	F	$\checkmark $	$\checkmark $	Multi-component weight management programmes.	FP/Txt		RevMan
PH53	Evidence review 1b	E	$\checkmark $	$\checkmark $	$\checkmark $	BOCF weight change	MD	MA	R	$\checkmark $	$\checkmark $	Multi-component weight management programmes.	FP/Txt/T		nr
PH53	Evidence review 1c	E	$\checkmark $	$\checkmark $	$\checkmark $	BOCF weight change	MD	MA	R	$\checkmark $	$\checkmark $	Multi-component weight management programmes.	FP/Txt/T		nr
PH48	Review 2 – Effectiveness of smoking cessation interventions in acute and maternity services	E	$\checkmark $	$\checkmark $	$\checkmark $	Abstinence from smoking at least six months after the start of the intervention	OR	MA	F				FP/Txt		RevMan
PH48	Review 3 – Barriers & facilitators for smoking cessation interventions in acute and maternity services	E	$\checkmark $		$\checkmark $	Impact of stopping smoking shortly before surgery on surgery outcomes / complications	RR	MA	R				FP/Txt		RevMan
PH48	Review 4 – Effectiveness of smoking cessation interventions in mental health services	E	$\checkmark $	$\checkmark $	$\checkmark $	Participants who made successful quit attempts	OR	MA	R	$\checkmark $	$\checkmark $	Pharmacological, psychological, be- havioural, or self-help intervention components	FP/Txt	$\checkmark $	RevMan
PH47	Review of effectiveness and cost effectiveness	E, CE	$\checkmark $	$\checkmark $	$\checkmark $	BMI, Zbmi	SMD	MA	R	$\checkmark $	$\checkmark $	Diet, physical activity, behaviour change inter- vention components.	FP/Txt	$\checkmark $	Stata
PH44	Review of effectiveness and barriers and facilitators	E	$\checkmark $		$\checkmark $	Self-reported physical activity	Mean, RR	MA	R				FP/Txt		RevMan
PH38	Evidence reviews	E, CE, DM	$\checkmark $	$\checkmark $	$\checkmark $	Progression to T2 Diabetes	Other	MA	R	$\checkmark $			FP		RevMan
PH38	R2 systematic review and meta-analysis of lifestyle, pharmacological and surgical interventions	E	$\checkmark $	$\checkmark $	$\checkmark $	Progression to T2 Diabetes	HR	MA and NMA	R	$\checkmark $	$\checkmark $	Lifestyle, drug, and sur- gical intervention com- ponents.	FP/Txt		RevMan

**Notation:** E: effectiveness, CE: cost-effectiveness, DM: decision model, RR: risk ratio, MD: mean difference, OR: odds ratio, SMD: standardised mean difference, HR: hazard ratio, MA: meta-analysis, NMA: network meta-analysis, nr: not reported, R: random effects, F: fixed effect, Txt: text, T: table, FP: forest plot

An evaluation of the intervention information reported in the reviews concluded that 12 (50%) reviews had lumped multiple (more than two) different interventions into a control versus intervention pairwise meta-analysis. Eleven (46%) of the reviews evaluated interventions that are made up of multiple components (e.g. interventions for preventing obesity in PH47 were made up of diet, physical activity and behavioural change components).

21 (88%) of the reviews presented the results of the meta-analysis in the form of a forest plot and 22 (92%) presented the results in the text of the report. 20 (83%) of the reviews used two or more forms of presentation for the results. Only three (13%) reviews assessed publication bias. The most common software to perform meta-analysis was RevMan in 14 (58%) of the reviews.

#### Reasons for not using meta-analytic methods

**Fig. 1:**
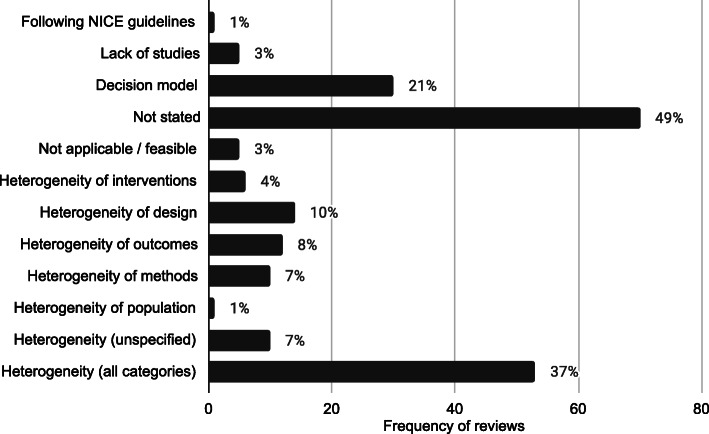
Frequency and proportions of reasons reported for not using statistical methods in quantitative evidence synthesis in NICE PH intervention reviews

The 143 reviews of effectiveness and cost effectiveness that did not use meta-analysis methods to synthesise the quantitative effectiveness evidence were searched for reasons behind this decision. 70 reports (49%) did not give a reason for not synthesising the data using a meta-analysis and 164 reasons were reported which are displayed in Fig. 1. Out of the remaining reviews, multiple reasons for not using a meta-analysis were given. 53 (37%) of the reviews reported at least one reason due to heterogeneity. 30 (21%) decision model reports did not give a reason and these are categorised separately. 5 (3%) reviews reported that meta-analysis was not applicable or feasible, 1 (1%) reported that they were following NICE guidelines and 5 (3%) reported that there were a lack of studies.

**Fig. 2:**
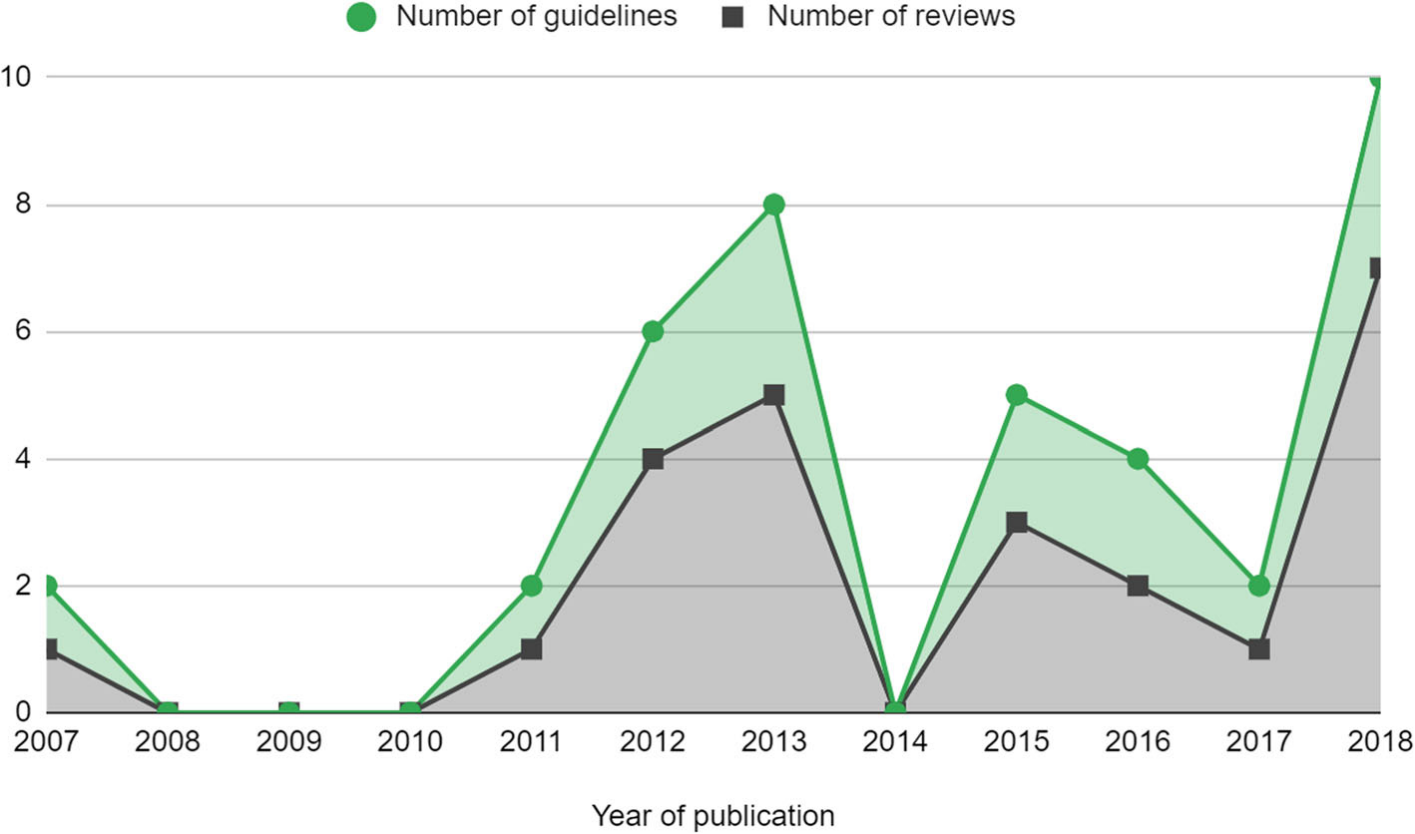
Number of meta-analyses in NICE PH guidelines by year. Guidelines that were published before 2012 had been updated since the previous review by Achana et al. (2014) [3]

The frequency of reviews and guidelines that used meta-analytic methods were plotted against year of publication, which is reported in Fig. 2. This showed that the number of reviews that used meta-analysis were approximately constant but there is some suggestion that the number of meta-analyses used per guideline increased, particularly in 2018.

#### Comparison of meta-analysis characteristics to original review

Table 4 compares the characteristics of the meta-analyses used in the evidence synthesis of NICE public health intervention guidelines to the original review by Achana et al. (2014) [3]. Overall, the characteristics in the updated review have not much changed from those in the original. These changes demonstrate that the use of meta-analysis in NICE guidelines has increased but remains low. Lumping of interventions still appears to be common in 50% of reviews. The implications of this are discussed in the next section.

**Table 4:** Meta-analysis characteristics: comparison to original review

	Original Review (39 guidelines)	Updated Review (24 Reviews)
RCTs only	4 (40%)	12 (50%)
Final outcomes	6 (60%)	20 (83%)
Lumping of interventions	7 (70%)	12 (50%)
Random effects meta-analysis	8 (80%)	19 (79%)
Fixed effects meta-analysis	1 (10%)	2 (8%)
Forest plots for presentation	9 (90%)	21 (88%)
Assessed publication bias	1 (10%)	3 (13%)

## Application of evidence synthesis methodology in a public health intervention: motivating example

Since the original review, evidence synthesis methods have been developed and can address some of the challenges of synthesising quantitative effectiveness evidence of public health interventions. Despite this, the previous section shows that the uptake of these methods is still low in NICE public health guidelines - usually limited to a pairwise meta-analysis.

It has been shown in the results above and elsewhere [12] that heterogeneity is a common reason for not synthesising the quantitative effectiveness evidence available from systematic reviews in public health. Statistical heterogeneity is the variation in the intervention effects between the individual studies. Heterogeneity is problematic in evidence synthesis as it leads to uncertainty in the pooled effect estimates in a meta-analysis which can make it difficult to interpret the pooled results and draw conclusions. Rather than exploring the source of the heterogeneity, often in public health intervention appraisals a random effects model is fitted which assumes that the study intervention effects are not equivalent but come from a common distribution [13, 14]. Alternatively, as demonstrated in the review update, heterogeneity is used as a reason to not undertake any quantitative evidence synthesis at all.

Since the size of the intervention effects and the methodological variation in the studies will affect the impact of the heterogeneity on a meta-analysis, it is inappropriate to base the methodological approach of a review on the degree of heterogeneity, especially within public health intervention appraisal where heterogeneity seems inevitable. Ioannidis et al. (2008) argued that there are ‘almost always’ quantitative synthesis options that may offer some useful insights in the presence of heterogeneity, as long as the reviewers interpret the findings with respect to their limitations [12].

In this section current evidence synthesis methods are applied to a motivating example in public health. This aims to demonstrate that methods beyond pairwise meta-analysis can provide appropriate and pragmatic information to public health decision makers to enable more informed decision making.

**Fig. 3:**
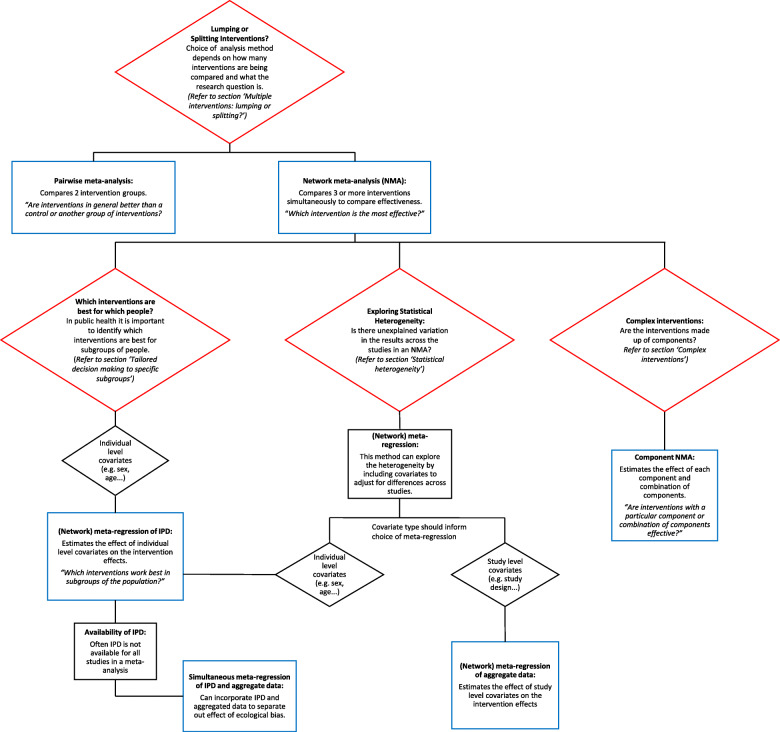
Summary of challenges that are faces in the evidence synthesis of public health interventions and methods that are discussed to overcome these challenges

Figure 3 summarises the narrative of this part of the paper and illustrates the methods that are discussed. The red boxes represent the challenges in synthesising quantitative effectiveness evidence and refers to the section within the paper for more detail. The blue boxes represent the methods that can be applied to investigate each challenge.

### Evaluating the effect of interventions for promoting the safe storage of cleaning products to prevent childhood poisoning accidents

To illustrate the methodological developments, a motivating example is used from the five year, NIHR funded, Keeping Children Safe Programme [15]. The project included a Cochrane systematic review that aimed to increase the use of safety equipment to prevent accidents at home in children under five years old. This application is intended to be illustrative of the benefits of new evidence synthesis methods since the previous review. It is not a complete, comprehensive analysis as it only uses a subset of the original dataset and therefore the results are not intended to be used for policy decision making. This example has been chosen as it demonstrates many of the issues in synthesising effectiveness evidence of public health interventions, including different study designs (randomised controlled trials, observational studies and cluster randomised trials), heterogeneity of populations or settings, incomplete individual participant data and complex interventions that contain multiple components.

This analysis will investigate the most effective promotional interventions for the outcome of ‘safe storage of cleaning products’ to prevent childhood poisoning accidents. There are 12 studies included in the dataset, with IPD available from nine of the studies. The covariate, single parent family, is included in the analysis to demonstrate the effect of being a single parent family on the outcome. In this example, all of the interventions are made up of one or more of the following components: education (Ed), free or low cost equipment (Eq), home safety inspection (HSI), and installation of safety equipment (In). A Bayesian approach using WinBUGS was used and therefore credible intervals (CrI) are presented with estimates of the effect sizes [16].

The original review paper by Achana et al. (2014) demonstrated pairwise meta-analysis and meta-regression using individual and cluster allocated trials, subgroup analyses, meta-regression using individual participant data (IPD) and summary aggregate data and NMA. This paper firstly applies NMA to the motivating example for context, followed by extensions to NMA.

### Multiple interventions: lumping or splitting?

Often in public health there are multiple intervention options. However, interventions are often lumped together in a pairwise meta-analysis. Pairwise meta-analysis is a useful tool for two interventions or, alternatively in the presence of lumping interventions, for answering the research question: ‘are interventions in general better than a control or another group of interventions?’. However, when there are multiple interventions, this type of analysis is not appropriate for informing health care providers which intervention should be recommended to the public. ‘Lumping’ is becoming less frequent in other areas of evidence synthesis, such as for clinical interventions, as the use of sophisticated synthesis techniques, such as NMA, increases (Achana et al. 2014) but lumping is still common in public health.

NMA is an extension of the pairwise meta-analysis framework to more than two interventions. Multiple interventions that are lumped into a pairwise meta-analysis are likely to demonstrate high statistical heterogeneity. This does not mean that quantitative synthesis could not be undertaken but that a more appropriate method, NMA, should be implemented. Instead the statistical approach should be based on the research questions of the systematic review. For example, if the research question is ‘are any interventions effective for preventing obesity?’, it would be appropriate to perform a pairwise meta-analysis comparing every intervention in the literature to a control. However, if the research question is ‘which intervention is the most effective for preventing obesity?’, it would be more appropriate and informative to perform a network meta-analysis, which can compare multiple interventions simultaneously and identify the best one.

NMA is a useful statistical method in the context of public health intervention appraisal, where there are often multiple intervention options, as it estimates the relative effectiveness of three or more interventions simultaneously, even if direct study evidence is not available for all intervention comparisons. Using NMA can help to answer the research question ‘what is the effectiveness of each intervention compared to all other interventions in the network?’.

**Fig. 4:**
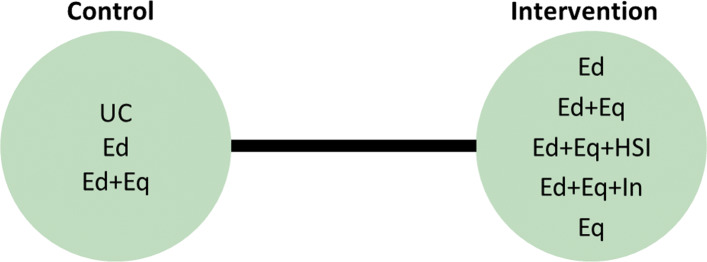
Network plot to illustrate how pairwise meta-analysis groups the interventions in the motivating dataset. **Notation** UC: Usual care, Ed: Education, Ed+Eq: Education and equipment, Ed+Eq+HSI: Education, equipment, and home safety inspection, Ed+Eq+In: Education, equipment and installation, Eq: Equipment

**Fig. 5:**
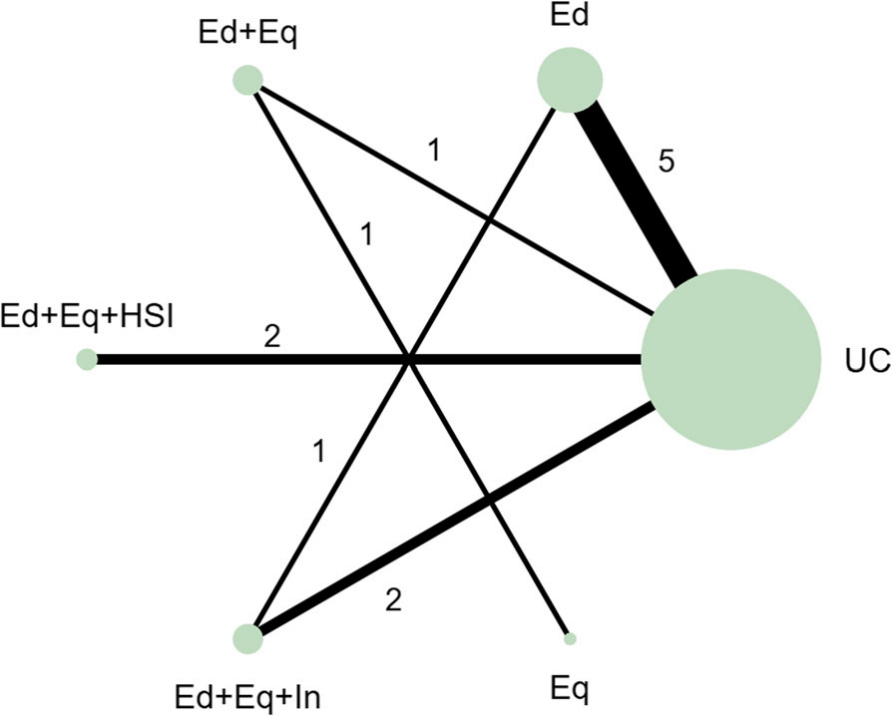
Network plot for the safe storage of cleaning products outcome. **Notation** UC: Usual care, Ed: Education, Ed+Eq: Education and equipment, Ed+Eq+HSI: Education, equipment, and home safety inspection, Ed+Eq+In: Education, equipment and installation, Eq: Equipment

In the motivating example there are six intervention options. The effect of lumping interventions is shown in Fig. 4, where different interventions in both the intervention and control arms are compared. There is overlap of intervention and control arms across studies and interpretation of the results of a pairwise meta-analysis comparing the effectiveness of the two groups of interventions would not be useful in deciding which intervention to recommend. In comparison, the network plot in Fig. 5 illustrates the evidence base of the prevention of childhood poisonings review comparing six interventions that promote the use of safety equipment in the home. Most of the studies use ‘usual care’ as a baseline and compare this to another intervention. There are also studies in the evidence base that compare pairs of the interventions, such as ‘Education and equipment’ to ‘Equipment’. The plot also demonstrates the absence of direct study evidence between many pairs of interventions, for which the associated treatment effects can be indirectly estimated using NMA.

An NMA was fitted to the motivating example to compare the six interventions in the studies from the review. The results are reported in the ‘triangle table’ in Table 5 [17]. The top right half of the table shows the direct evidence between pairs of the interventions in the corresponding rows and columns by either pooling the studies as a pairwise meta-analysis or presenting the single study results if evidence is only available from a single study. The bottom left half of the table reports the results of the NMA. The gaps in the top right half of the table arise where no direct study evidence exists to compare the two interventions. For example, there is no direct study evidence comparing ‘Education’ (Ed) to ‘Education, equipment and home safety inspection’ (Ed+Eq+HSI). The NMA, however, can estimate this comparison through the direct study evidence as an odds ratio of 3.80 with a 95% credible interval of (1.16, 12.44). The results suggest that the odds of safely storing cleaning products in the Ed+Eq+HSI intervention group is 3.80 times the odds in the Ed group. The results demonstrate a key benefit of NMA that all intervention effects in a network can be estimated using indirect evidence, even if there is no direct study evidence for some pairwise comparisons. This is based on the consistency assumption (that estimates of intervention effects from direct and indirect evidence are consistent) which should be checked when performing an NMA. This is beyond the scope of this paper and details on this can be found elsewhere [18].

**Table 5:** Results of an NMA expressed as odds ratios with 95% CrIs

	UC	Ed	Ed+Eq	Ed+Eq+HSI	Ed+Eq+In	Eq
**UC**	-	1.36 (0.93,1.98)	1.65 (0.87, 3.17)	2.90 (0.74, 11.33)	1.18 (0.96, 1.47)	
**Ed**	1.33 (0.79, 2.35)	-			1.41 (0.49, 4.06)	
**Ed+Eq**	1.68 (0.53, 5.43)	1.25 (0.35, 4.54)	-			0.32 (0.01, 7.96)
**Ed+Eq+HSI**	5.11 (1.83, 14.58)	3.80 (1.16, 12.44)	3.03 (0.63, 14.22)	-		
**Ed+Eq+In**	1.26 (0.70, 2.62)	0.96 (0.46, 2.15)	0.76 (0.21, 3.02)	0.25 (0.08, 0.90)	-	
**Eq**	0.30 (0.00, 10.75)	0.22 (0.00, 8.25)	0.19 (0.00, 5.27)	0.06 (0.00, 2.46)	0.23 (0.00, 8.51)	-

NMA results are in the bottom left half of the table. Pairwise meta-analysis or single study results, where no other direct evidence is available, are in the top right half of the table.

**Notation** UC: Usual care, Ed: Education, Ed+Eq: Education and equipment, Ed+Eq+HSI: Education, equipment, and home safety inspection, Ed+Eq+In: Education, equipment and installation, Eq: Equipment

NMA can also be used to rank the interventions in terms of their effectiveness and estimate the probability that each intervention is likely to be the most effective. This can help to answer the research question ‘which intervention is the best?’ out of all of the interventions that have provided evidence in the network. The rankings and associated probabilities for the motivating example are presented in Table 6. It can be seen that in this case the ‘education, equipment and home safety inspection’ (Ed+Eq+HSI) intervention is ranked first, with a 0.87 probability of being the best intervention. However, there is overlap of the 95% credible intervals of the median rankings. This overlap reflects the uncertainty in the intervention effect estimates and therefore it is important that the interpretation of these statistics clearly communicates this uncertainty to decision makers.

**Table 6:** Results of the NMA: probability that each intervention is the best and their ranks

Intervention	P(best)	Median rank (95% CrI)	Mean rank
Ed+Eq+HSI	0.87	1 (1, 3)	1.17
Ed+Eq	0.05	2 (1, 5)	2.89
Ed	0.01	3 (2, 6)	3.44
Ed+Eq+In	0.01	4 (2, 6)	3.63
UC	0.00	5 (3, 6)	4.87
Eq	0.06	6 (1, 6)	5.00

**Notation** P(best): probability that intervention is the best, CrI: Credible interval, UC: Usual care, Ed: Education, Ed+Eq: Education and equipment, Ed+Eq+HSI: Education, equipment, and home safety inspection, Ed+Eq+In: Education, equipment and installation, Eq: Equipment

NMA has the potential to be extremely useful but is underutilised in the evidence synthesis of public health interventions. The ability to compare and rank multiple interventions in an area where there are often multiple intervention options is invaluable in decision making for identifying which intervention to recommend. NMA can also include further literature in the analysis, compared to a pairwise meta-analysis, by expanding the network to improve the uncertainty in the effectiveness estimates.

### Statistical heterogeneity

When heterogeneity remains in the results of an NMA, it is useful to explore the reasons for this. Strategies for dealing with heterogeneity involve the inclusion of covariates in a meta-analysis or NMA to adjust for the differences in the covariates across studies [19]. Meta-regression is a statistical method developed from meta-analysis that includes covariates to potentially explain the between-study heterogeneity ‘with the aim of estimating treatment-covariate interactions’ (Saramago et al. 2012). NMA has been extended to network meta-regression which investigates the effect of trial characteristics on multiple intervention effects. Three ways have been suggested to include covariates in an NMA: single covariate effect, exchangeable covariate effects and independent covariate effects which are discussed in more detail in the NICE Technical Support Document 3 [14]. This method has the potential to assess the effect of study level covariates on the intervention effects, which is particularly relevant in public health due to the variation across studies.

The most widespread method of meta-regression uses study level data for the inclusion of covariates into meta-regression models. Study level covariate data is when the data from the studies are aggregated, e.g. the proportion of participants in a study that are from single parent families compared to dual parent families. The alternative to study level data is individual participant data (IPD), where the data are available and used as a covariate at the individual level e.g. the parental status of every individual in a study can be used as a covariate. Although IPD is considered to be the gold standard for meta-analysis, aggregated level data is much more commonly used as it is usually available and easily accessible from published research whereas IPD can be hard to obtain from study authors.

There are some limitations to network meta-regression. In our motivating example, using the single parent covariate in a meta-regression would estimate the relative difference in the intervention effects of a population that is made up of 100% single parent families compared to a population that is made up of 100% dual parent families. This interpretation is not as useful as the analysis that uses IPD, which would give the relative difference of the intervention effects in a single parent family compared to a dual parent family. The meta-regression using aggregated data would also be susceptible to ecological bias. Ecological bias is where the effect of the covariate is different at the study level compared to the individual level [14]. For example, if each study demonstrates a relationship between a covariate and the intervention but the covariate is similar across the studies, a meta-regression of the aggregate data would not demonstrate the effect that is observed within the studies [20].

Although meta-regression is a useful tool for investigating sources of heterogeneity in the data, caution should be taken when using the results of meta-regression to explain how covariates affect the intervention effects. Meta-regression should only be used to investigate study characteristics, such as the duration of intervention, which will not be susceptible to ecological bias and the interpretation of the results (the effect of intervention duration on intervention effectiveness) would be more meaningful for the development of public health interventions.

Since the covariate of interest in this motivating example is not a study characteristic, meta-regression of aggregated covariate data was not performed. Network meta-regression including IPD and aggregate level data was developed by Samarago et al. (2012) [21] to overcome the issues with aggregated data network meta-regression, which is discussed in the next section.

### Tailored decision making to specific sub-groups

In public health it is important to identify which interventions are best for which people. There has been a recent move towards precision medicine. In the field of public health the ‘concept of precision prevention may [...] be valuable for efficiently targeting preventive strategies to the specific subsets of a population that will derive maximal benefit’ (Khoury and Evans, 2015). Tailoring interventions has the potential to reduce the effect of inequalities in social factors that are influencing the health of the population. Identifying which interventions should be targeted to which subgroups can also lead to better public health outcomes and help to allocate scarce NHS resources. Research interest, therefore, lies in identifying participant level covariate-intervention interactions.

IPD meta-analysis uses data at the individual level to overcome ecological bias. The interpretation of IPD meta-analysis is more relevant in the case of using participant characteristics as covariates since the interpretation of the covariate-intervention interaction is at the individual level rather than the study level. This means that it can answer the research question: ‘which interventions work best in subgroups of the population?’. IPD meta-analyses are considered to be the gold standard for evidence synthesis since it increases the power of the analysis to identify covariate-intervention interactions and it has the ability to reduce the effect of ecological bias compared to aggregated data alone. IPD meta-analysis can also help to overcome scarcity of data issues and has been shown to have higher power and reduce the uncertainty in the estimates compared to analysis including only summary aggregate data [22].

Despite the advantages of including IPD in a meta-analysis, in reality it is often very time consuming and difficult to collect IPD for all of the studies [21]. Although data sharing is becoming more common, it remains time consuming and difficult to collect IPD for all studies in a review. This results in IPD being underutilised in meta-analyses. As an intermediate solution, statistical methods have been developed, such as the NMA in Samarago et al. (2012), that incorporates both IPD and aggregate data. Methods that simultaneously include IPD and aggregate level data have been shown to reduce uncertainty in the effect estimates and minimise ecological bias [20, 21]. A simulation study by Leahy et al. (2018) found that an increased proportion of IPD resulted in more accurate and precise NMA estimates [23].

An NMA including IPD, where it is available, was performed, based on the model presented in Samarago et al. (2012) [21]. The results in Table 7 demonstrates the detail that this type of analysis can provide to base decisions on. More relevant covariate-intervention interaction interpretations can be obtained, for example the regression coefficients for covariate-intervention interactions are the individual level covariate intervention interactions or the ‘within study interactions’ that are interpreted as the effect of being in a single parent family on the effectiveness of each of the interventions. For example, the effect of Ed+Eq compared to UC in a single parent family is 1.66 times the effect of Ed+Eq compared to UC in a dual parent family but this is not an important difference as the credible interval crosses 1. The regression coefficients for the study level covariate-intervention interactions or the ‘between study interactions’ can be interpreted as the relative difference in the intervention effects of a population that is made up of 100% single parent families compared to a population that is made up of 100% dual parent families.

**Table 7:** Results of network meta-regression including IPD and summary aggregate data

Parameter	Intervention		OR (95% CrI)
Intervention effects		Ed	0.95 (0.10, 5.09)
(compared to UC)		Ed+Eq	1.46 (0.16, 10.61)
		Ed+Eq+HSI	2.30 (0.47, 11.88)
		Ed+Eq+In	1.05 (0.12, 6.68)
		Eq	49.84 (0.14, 1.84 ×10^6^)
Regression coefficients for	Within study interactions	Ed	0.98 (0.44, 2.02)
covariate-intervention		Ed+Eq	1.66 (0.64, 20.68)
interactions (compared to		Ed+Eq+HSI	1.16 (0.39, 4.92)
UC)		Ed+Eq+In	1.04 (0.65, 1.67)
		Eq	1.15 (0.15, 15.38)
	Between study interactions	Ed	2.22 (0.13, 158.00)
		Ed+Eq	2.42 (0.04, 278.40)
		Ed+Eq+HSI	2.34 (0.07, 180.50)
		Ed+Eq+In	2.31 (0.04, 396.3)
		Eq	2.40 (0.04, 310.90)
Heterogeneity estimates		Between study variance	0.64 (0.05, 3.07)
		Within study interaction variance	0.33 (0.00, 3.76)
		Between study interaction variance	0.80 (0.00, 3.76)

**Notation** CrI: Credible interval, UC: Usual care, Ed: Education, Ed+Eq: Education and equipment, Ed+Eq+HSI: Education, equipment, and home safety inspection, Ed+Eq+In: Education, equipment and installation, Eq: Equipment

### Complex interventions

**Fig. 6:**
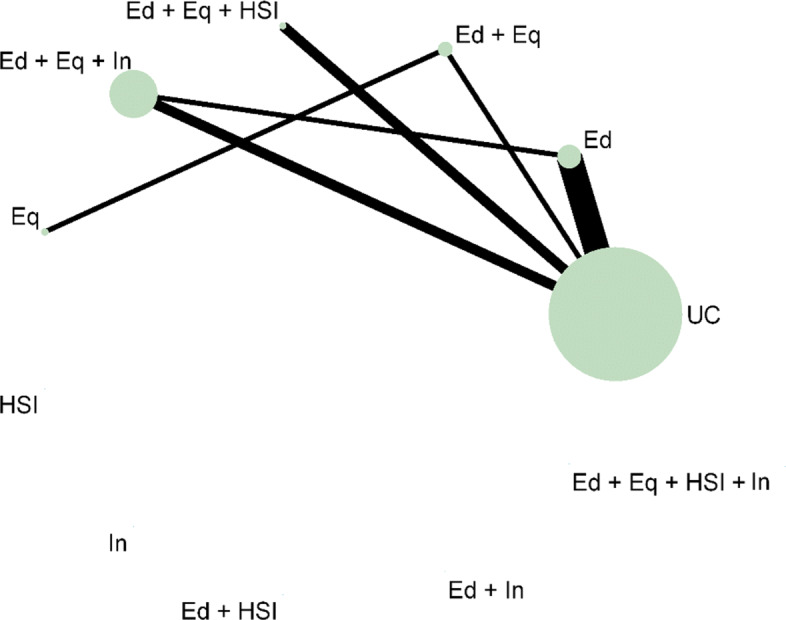
Network plot that illustrates how component network meta-analysis can estimate the effectiveness of intervention components and combinations of components, even when they are not included in the direct evidence. **Notation** UC: Usual care, Ed: Education, Eq: Equipment, Installation, Ed+Eq: Education and equipment, Ed+HSI: Education and home safety inspection, Ed+In: Education and installation, Eq+HSI: Equipment and home safety inspection, Eq+In: equipment and installation, HSI+In: Home safety inspection and installation, Ed+Eq+HSI: Education, equipment, and home safety inspection, Ed+Eq+In: Education, equipment and installation, Eq+HSI+In: Equipment, home safety inspection and installation, Ed+Eq+HSI+In: Education, equipment, home safety inspection and installation

In many public health research settings the complex interventions are comprised of a number of components. An NMA can compare all of the interventions in a network as they are implemented in the original trials. However, NMA does not tell us which components of the complex intervention are attributable to this effect. It could be that particular components, or the interacting effect of multiple components, are driving the effectiveness and other components are not as effective. Often, trials have not directly compared every combination of components as there are so many component combination options, it would be inefficient and impractical. Component NMA was developed by Welton et al. (2009) to estimate the effect of each component of the complex interventions and combination of components in a network, in the absence of direct trial evidence and answers the question: ‘are interventions with a particular component or combination of components effective?’ [11]. For example, for the motivating example, in comparison to Fig. 5, which demonstrates the interventions that an NMA can estimate effectiveness, Fig. 6 demonstrates all of the possible interventions of which the effectiveness can be estimated in a component NMA, given the components present in the network.

The results of the analyses of the main effects, two way effects and full effects models are shown in Table 8. The models, proposed in the original paper by Welton et al. (2009), increase in complexity as the assumptions regarding the component effects relax [24]. The main effects component NMA assumes that the components in the interventions each have separate, independent effects and intervention effects are the sum of the component effects. The two-way effects models assumes that there are interactions between pairs of the components, so the effects of the interventions are more than the sum of the effects. The full effects model assumes that all of the components and combinations of the components interact. Component NMA did not provide further insight into which components are likely to be the most effective since all of the 95% credible intervals were very wide and overlapped 1. There is a lot of uncertainty in the results, particularly in the 2-way and full effects models. A limitation of component NMA is that there are issues with uncertainty when data is scarce. However, the results demonstrate the potential of component NMA as a useful tool to gain better insights from the available dataset.

**Table 8:** Results of the complex interventions analysis. All results are presented as OR (95% CrI)

Intervention component combination	Main effects model	Two way effects model	Full effects model
Ed	1.35 (0.83, 2.34)	1.33 (0.79, 2.35)	1.32 (0.79, 2.32)
Eq	1.24 (0.36, 4.02)	0.35 (0.00, 11.41)	0.34 (0.00, 12.08)
HSI	3.04 (0.68, 13.41)	0.71 (0.00, 6.06 ×10^6^)	2.24 (0.00, 6.67 ×10^7^)
In	0.76 (0.23, 2.91)	0.94 (0.00, 8.29 ×10^6^)	0.95 (0.00, 2.12 ×10^7^)
Ed+Eq		3.54 (0.13, 984.20)	3.59 (0.12, 658.30)
Ed+HSI		1.30 (0.00, 5.53 ×10^6^)	1.04 (0.00, 2.18 ×10^7^)
Ed+In		0.89 (0.00, 7.97 ×10^6^)	0.96 (0.00, 2.04 ×10^7^)
Eq+HSI		1.68 (0.00, 2.84 ×10^6^)	0.88 (0.00, 1.22 ×10^7^)
Eq+In		0.94 (0.00, 7.31 ×10^6^)	0.90 (0.00, 2.21 ×10^7^)
HSI+In		0.95 (0.00, 2.77 ×10^8^)	0.99 (0.00, 3.66 ×10^8^)
Ed+Eq+HSI			1.35 (0.00, 2.49 ×10^7^)
Ed+Eq+In			0.95 (0.00, 2.66 ×10^7^)
Eq+HSI+In			0.94 (0.00, 3.15 ×10^8^)
Ed+Eq+HSI+In			0.93 (0.00, 3.28 ×10^8^)
*τ*^2^	0.07 (0.00, 1.08)	0.08 (0.00, 1.21)	0.08 (0.00, 1.24)

**Notation** CrI: Credible interval, UC: Usual care, Ed: Education, Eq: Equipment, Installation, Ed+Eq: Education and equipment, Ed+HSI: Education and home safety inspection, Ed+In: Education and installation, Eq+HSI: Equipment and home safety inspection, Eq+In: equipment and installation, HSI+In: Home safety inspection and installation, Ed+Eq+HSI: Education, equipment, and home safety inspection, Ed+Eq+In: Education, equipment and installation, Eq+HSI+In: Equipment, home safety inspection and installation, Ed+Eq+HSI+In: Education, equipment, home safety inspection and installation, *τ*^2^: between study variance

In practice, this method has rarely been used since its development [24–26]. It may be challenging to define the components in some areas of public health where many interventions have been studied. However, the use of meta-analysis for planning future studies is rarely discussed and component NMA would provide a useful tool for identifying new component combinations that may be more effective [27]. This type of analysis has the potential to prioritise future public health research, which is especially useful where there are multiple intervention options, and identify more effective interventions to recommend to the public.

### Further methods / other outcomes

The analysis and methods described in this paper only cover a small subset of the methods that have been developed in meta-analysis in recent years. Methods that aim to assess the quality of evidence supporting a NMA and how to quantify how much the evidence could change due to potential biases or sampling variation before the recommendation changes have been developed [28, 29]. Models adjusting for baseline risk have been developed to allow for different study populations to have different levels of underlying risk, by using the observed event rate in the control arm [30, 31]. Multivariate methods can be used to compare the effect of multiple interventions on two or more outcomes simultaneously [32]. This area of methodological development is especially appealing within public health where studies assess a broad range of health effects and typically have multiple outcome measures. Multivariate methods offer benefits over univariate models by allowing the borrowing of information across outcomes and modelling the relationships between outcomes which can potentially reduce the uncertainty in the effect estimates [33]. Methods have also been developed to evaluate interventions with classes or different intervention intensities, known as hierarchical interventions [34]. These methods were not demonstrated in this paper but can also be useful tools for addressing challenges of appraising public health interventions, such as multiple and surrogate outcomes.

This paper only considered an example with a binary outcome. All of the methods described have also been adapted for other outcome measures. For example, the Technical Support Document 2 proposed a Bayesian generalised linear modelling framework to synthesise other outcome measures. More information and models for continuous and time-to-event data is available elsewhere [21, 35–38].

## Software and guidelines

In the previous section, meta-analytic methods that answer more policy relevant questions were demonstrated. However, as shown by the update to the review, methods such as these are still under-utilised. It is suspected from the NICE public health review that one of the reasons for the lack of uptake of methods in public health could be due to common software choices, such as RevMan, being limited in their flexibility for statistical methods.

Table 9 provides a list of software options and guidance documents that are more flexible than RevMan for implementing the statistical methods illustrated in the previous section to make these methods more accessible to researchers.

**Table 9:** Software for fitting meta-analysis models (full references in bibliography)

Method	Software options	Additional guidance (authors and reference number)
Network Plots	Stata: networkplotfrom the mvmeta package [39]	Chaimani et al. (2013) [40]
	R: netgraph command in netmeta [41], gemtc [42], pcnetmeta [43]	
		Rucker and Schwarzer (2016) [44]
Network Meta-Analysis	WinBUGS: flexible modelling framework, model code available from the Univerisity of Bristol website (https://www.bristol. ac.uk/population-health-sciences/ centres/cresyda/mpes/code/).	Welton et al. (2012) [45], Dias and Caldwell (2019) [17]
		Dias et al. [46]
	R2WinBUGS [47], BUGSnet [48]	
	R: netgraph command in netmeta [41], gemtc [42], pcnetmeta [43]	[27], Neupane et al. (2014) [49]
	Stata: mvmeta package [50]	Chaimani and Salanti (2015) [51], Chaimani et al. (2013) [40]
	Webtools: MetaInsight [52], MetaDTA [53] and CINeMA [54]	
Network Meta-Regression	WinBUGS: can utilise study level covariates from NMA models, model code available from the Univerisity of Bristol website (https://www.bristol. ac.uk/population-health-sciences/ centres/cresyda/mpes/code/).	
	Stata: metareg command, mvmeta package	
	R: mvmeta package, metafor package [55], GeMTC package [42]	
IPD Meta-Analysis	WinBUGS: inclusion of IPD and aggregated data model available in paper by Saramago et al. (2012) [21]	Freeman et al. (2018) [24], Freeman and Carpenter (2017) [56], Riley et al. (2008) [57]
		Debray et al. (2015) [58], Tierney et al. (2015) [59]
		PRISMA-IPD checklist [60]
Component Network Meta-Analysis	WinBUGS: component NMA model code available from the Univerisity of Bristol website (https://www.bristol. ac.uk/population-health-sciences/ centres/cresyda/mpes/code/).	Welton et al. (2009) [11]
	R: The additive model can be implemented using the discomb command in the frequentist netmeta package [41]	
		Higgins et al. (2019) [2], Caldwell and Welton (2016) [5]
		Melendez-Torres et al. (2015) [6]
		Inclusion of covariates in component NMA models by Freeman et al. (2018) [24]

In this paper, the network plot in Figs. 5 and 6 were produced using the networkplot command from the mvmeta package [39] in Stata [61]. WinBUGS was used to fit the NMA in this paper by adapting the code in the book ‘Evidence Synthesis for Decision Making in Healthcare’ which also provides more detail on Bayesian methods and assessing convergence of Bayesian models [45]. The model for including IPD and summary aggregate data in an NMA was based on the code in the paper by Saramago et al. (2012). The component NMA in this paper was performed in WinBUGS through R2WinBUGS, [47] using the code in Welton et al. (2009) [11].

WinBUGS is a flexible tool for fitting complex models in a Bayesian framework. The NICE Decision Support Unit produced a series of Evidence Synthesis Technical Support Documents [46] that provide a comprehensive technical guide to methods for evidence synthesis and WinBUGS code is also provided for many of the models. Complex models can also be performed in a frequentist framework. Code and commands for many models are available in R and STATA (see Table 9).

The software, R2WinBUGS, was used in the analysis of the motivating example. Increasing numbers of researchers are using R and so packages that can be used to link the two softwares by calling BUGS models in R, packages such as R2WinBUGS, can improve the accessibility of Bayesian methods [47]. The new R package, BUGSnet, may also help to facilitate the accessibility and improve the reporting of Bayesian NMA [48]. Webtools have also been developed as a means of enabling researchers to undertake increasingly complex analyses [52, 53]. Webtools provide a user-friendly interface to perform statistical analyses and often help in the reporting of the analyses by producing plots, including network plots and forest plots. These tools are very useful for researchers that have a good understanding of the statistical methods they want to implement as part of their review but are inexperienced in statistical software.

## Discussion

This paper has reviewed NICE public health intervention guidelines to identify the methods that are currently being used to synthesise effectiveness evidence to inform public health decision making. A previous review from 2012 was updated to see how method utilisation has changed. Methods have been developed since the previous review and these were applied to an example dataset to show how methods can answer more policy relevant questions. Resources and guidelines for implementing these methods were signposted to encourage uptake.

The review found that the proportion of NICE guidelines containing effectiveness evidence summarised using meta-analysis methods has increased since the original review, but remains low. The majority of the reviews presented only narrative summaries of the evidence - a similar result to the original review. In recent years, there has been an increased awareness of the need to improve decision making by using all of the available evidence. As a result, this has led to the development of new methods, easier application in standard statistical software packages, and guidance documents. Based on this, it would have been expected that their implementation would rise in recent years to reflect this, but the results of the review update showed no such increasing pattern.

A high proportion of NICE guideline reports did not provide a reason for not applying quantitative evidence synthesis methods. Possible explanations for this could be time or resource constraints, lack of statistical expertise, being unaware of the available methods or poor reporting. Reporting guidelines, such as the Preferred Reporting Items for Systematic Reviews and Meta-Analyses (PRISMA), should be updated to emphasise the importance of documenting reasons for not applying methods, as this can direct future research to improve uptake.

Where it was specified, the most common reported reason for not conducting a meta-analysis was heterogeneity. Often in public health, the data is heterogeneous due to the differences between studies in population, design, interventions or outcomes. A common misconception is that the presence of heterogeneity implies that it is not possible to pool the data. Meta-analytic methods can be used to investigate the sources of heterogeneity, as demonstrated in the NMA of the motivating example, and the use of IPD is recommended where possible to improve the precision of the results and reduce the effect of ecological bias. Although caution should be exercised in the interpretation of the results, quantitative synthesis methods provide a stronger basis for making decisions than narrative accounts because they explicitly quantify the heterogeneity and seek to explain it where possible.

The review also found that the most common software to perform the synthesis was RevMan. RevMan is very limited in its ability to perform advanced statistical analyses, beyond that of pairwise meta-analysis, which might explain the above findings. Standard software code is being developed to help make statistical methodology and application more accessible and guidance documents are becoming increasingly available.

The evaluation of public health interventions can be problematic due to the number and complexity of the interventions. NMA methods were applied to a real Cochrane public health review dataset. The methods that were demonstrated showed ways to address some of these issues, including the use of NMA for multiple interventions, the inclusion of covariates as both aggregated data and IPD to explain heterogeneity, and the extension to component network meta-analysis for guiding future research. These analyses illustrated how the choice of synthesis methods can enable more informed decision making by allowing more distinct interventions, and combinations of intervention components, to be defined and their effectiveness estimated. It also demonstrated the potential to target interventions to population subgroups where they are likely to be most effective. However, the application of component NMA to the motivating example has also demonstrated the issues around uncertainty if there are a limited number of studies observing the interventions and intervention components.

The application of methods to the motivating example demonstrated a key benefit of using statistical methods in a public health context compared to only presenting a narrative review – the methods provide a quantitative estimate of the effectiveness of the interventions. The uncertainty from the credible intervals can be used to demonstrate the lack of available evidence. In the context of decision making, having pooled estimates makes it much easier for decision makers to assess the effectiveness of the interventions or identify when more research is required. The posterior distribution of the pooled results from the evidence synthesis can also be incorporated into a comprehensive decision analytic model to determine cost-effectiveness [62]. Although narrative reviews are useful for describing the evidence base, the results are very difficult to summarise in a decision context.

Although heterogeneity seems to be inevitable within public health interventions due to their complex nature, this review has shown that it is still the main reported reason for not using statistical methods in evidence synthesis. This may be due to guidelines that were originally developed for clinical treatments that are tested in randomised conditions still being applied in public health settings. Guidelines for the choice of methods used in public health intervention appraisals could be updated to take into account the complexities and wide ranging areas in public health. Sophisticated methods may be more appropriate in some cases than simpler models for modelling multiple, complex interventions and their uncertainty, given the limitations are also fully reported [19]. Synthesis may not be appropriate if statistical heterogeneity remains after adjustment for possible explanatory covariates but details of exploratory analysis and reasons for not synthesising the data should be reported. Future research should focus on the application and dissemination of the advantages of using more advanced methods in public health, identifying circumstances where these methods are likely to be the most beneficial, and ways to make the methods more accessible, for example, the development of packages and web tools.

## Conclusions

There is an evident need to facilitate the translation of the synthesis methods into a public health context and encourage the use of methods to improve decision making. This review has shown that the uptake of statistical methods for evaluating the effectiveness of public health interventions is slow, despite advances in methods that address specific issues in public health intervention appraisal and the publication of guidance documents to complement their application.

## Supplementary Information

**Additional file 1** Key for the Nice public health guideline codes. Available in *NICEGuidelinesKey.xlsx*.

**Additional file 2** NICE public health intervention guideline review flowchart for the inclusion and exclusion of documents. Available in *Flowchart.JPG*.

## Data Availability

The dataset supporting the conclusions of this article is included within the article.

## Abbreviations

NICE: National institute for health and care excellence
NMA: Network meta-analysis
IPD: Individual participant data
Ed: Education
HSI: Home safety inspection
In: Installation
Eq: Equipment
CrI: Credible interval
PRISMA: Preferred reporting items for systematic reviews and meta-analyses
