# Supplementary figures and images for: A review of the quantitative effectiveness evidence synthesis methods used in public health intervention guidelines
Source: BMC Public Health. 2021 Feb 3;21:278. doi: 10.1186/s12889-021-10162-8 (PMC7860217; doi:10.1186/s12889-021-10162-8)

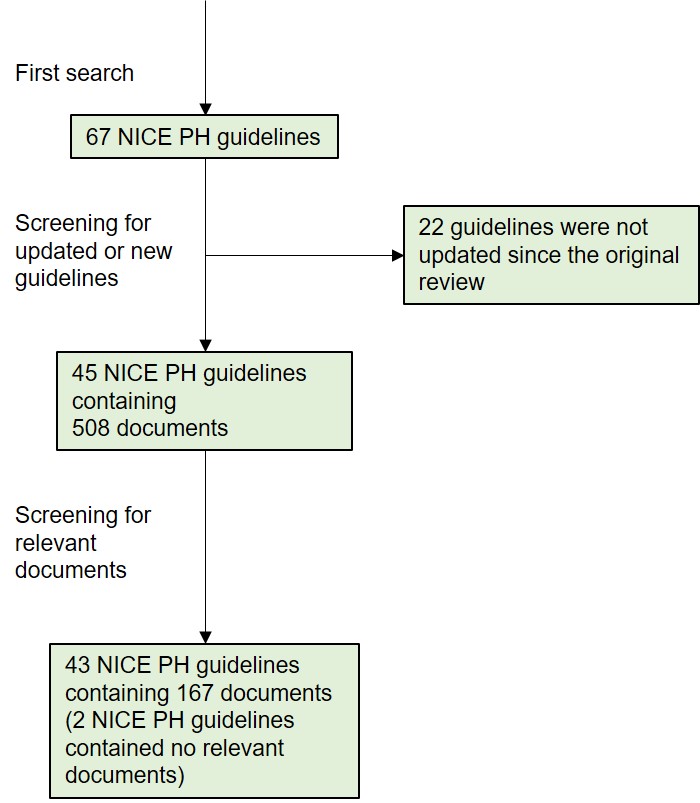

Supplement: Supplementary file 2 — Additional file 2 NICE public health intervention guideline review flowchart for the inclusion and exclusion of documents. Available in Flowchart.JPG. [file 12889_2021_10162_MOESM2_ESM.jpg]
